# Is It an Outbreak of Health Care-Associated Infection? An Investigation of Binocular Conjunctival Congestion After Laparoscopic Cholecystectomy Was Traced to Chitosan Derivatives

**DOI:** 10.3389/fmed.2022.759945

**Published:** 2022-03-07

**Authors:** Sidi Liu, Xun Huang, Chenchao Fu, Qingya Dou, Jie Li, Xuelian Feng, Yang Mo, Xiujuan Meng, Cui Zeng, Anhua Wu, Chunhui Li

**Affiliations:** ^1^Infection Control Center, Xiangya Hospital of Central South University, Changsha, China; ^2^National Clinical Research Center for Geriatric Disorders, Xiangya Hospital of Central South University, Changsha, China; ^3^Operating Room Department, Xiangya Hospital of Central South University, Changsha, China; ^4^Day Ward Unit, Xiangya Hospital of Central South University, Changsha, China

**Keywords:** health care-associated infection (HAI), conjunctival congestion, nosocomial infection prevention and control, biological material, regulation

## Abstract

**Background:**

From May 6 to May 23, 2019, 24 (80.00%) patients who underwent laparoscopic cholecystectomy (LC) developed binocular conjunctival congestion within 4–8 h after their operation in the day ward of a teaching hospital.

**Methods:**

Nosocomial infection prevention and control staff undertook procedural and environmental investigations, performed a case-control retrospective study (including 24 cases and 48 controls), and reviewed all lot numbers of biological material products to investigate the suspected outbreak of health care-associated infection.

**Findings:**

Initially, an outbreak of health care-associated infection caused by bacteria was hypothesized. We first suspected the membranes that covered patients' eyes were cut using non-sterile scissors and thus contaminated, but they failed to yield bacteria. In addition, both corneal and conjunctival fluorescein staining results were negative in case-patients and isolated bacteria were ubiquitous in the environment or common skin commensals or normal flora of conjunctiva from 218 samples from day surgery and the day ward. Hence, we considered a non-infectious factor as the most likely cause of the binocular conjunctival congestion. Then, we found that case-patients were more likely than LC surgery patients without binocular conjunctival congestion to be exposed to biological materials in a retrospective case-control study. When we reviewed lot numbers, duration of use, and the number of patients who received four biological material products during LC in the day ward, we found that the BLK1821 lot of a modified chitosan medical membrance (the main ingredient is chitosan, a linear cationic polysaccharide) was used concurrently to when the case aggregation appeared. Finally, we surmised there was a correlation between this product and the outbreak of binocular conjunctival congestion. Relapse of the pseudo-outbreak has not been observed since stopping usage of the product for 6 months.

**Conclusion:**

A cluster of binocular non-infectious conjunctival congestion diagnosed after LC proved to be a pseudo-outbreak. We should pay more attention to adverse events caused by biomaterials in hospitals.

## Introduction

Health care-associated infections (HAIs) are a major global public health problem that can prolong hospital stays, create long-term disability, increase patient morbidity and mortality rates, and place a massive additional financial burden on health systems and families alike (1), especially in developing countries (2). Outbreaks of HAI are not uncommon in hospital settings, and various pathogens may be responsible (3). Signs of a suspected outbreak could stem from the identification of a cluster of cases (infection or colonization), the detection of abnormal pathogens or antibiotics-resistance mechanisms, or even the observation of serious infection control violations. Contamination of hospital linens (4, 5), water distribution systems (6, 7), and the faucet aerator (8) have been reported to cause HAI outbreaks; however, outbreaks of unexplained HAIs also occur and require further investigation. Rapid identification of the source of HAI outbreaks is crucial to eliminate the immediate risk and prevent future harm to patients (9). Timely public reporting of these events can help other hospitals identify and mitigate risks (10).

Conjunctival congestion and redness can be caused by infectious causes, such as related bacteria or viruses. Typically, we may consider an HAI outbreak to be possible if several patients in the same ward experience conjunctival congestion and redness within a short period of time. However, sometimes when we deal with suspected HAI outbreaks, we may find other causes. In a day ward of our hospital, a cluster of patients who underwent laparoscopic cholecystectomy (LC) developed binocular conjunctival congestion within a short period. Initially, we thought it might be an HAI outbreak caused by bacteria. However, an unexpected and often-overlooked non-infectious factor caused by chitosan was found, and we herein report it internationally for the first time. In this study, we describe the investigation process, results, and subsequent disposition of a cluster of binocular non-infectious conjunctival congestion cases that appeared after LC.

## Methods

### Background

**Figure 1:**
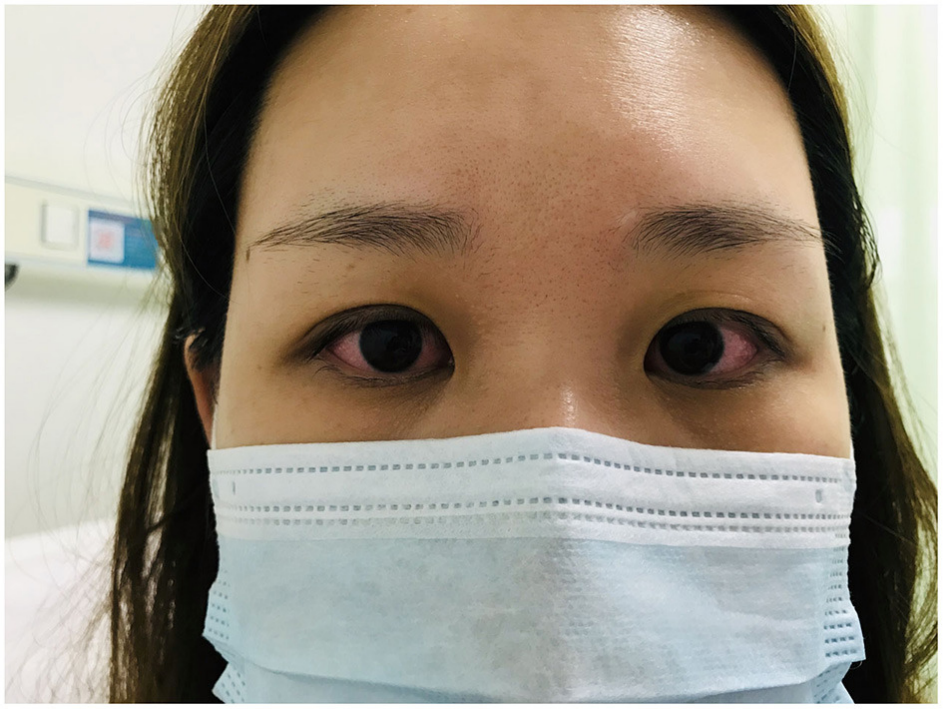
Signs of binocular conjunctival congestion in a female patient who underwent LC. This patient presented with binocular conjunctival congestion but without secretions, tearing, itching, pain, burning/stinging, photophobia, foreign body sensation, eye discomfort, or decreased vision within 6 h.

On May 9, 2019, the nosocomial infection prevention and control (IPC) department was notified by clinicians that nine (9/11, 81.82%) patients who had undergone LC developed binocular conjunctival congestion (Figure 1) postoperatively within 4–8 h after their surgery in a day ward of Xiangya Hospital of Central South University, a tertiary hospital in China with 3,000 beds, between May 6 and May 8, 2019. The IPC team subsequently accessed electronic medical records and performed a retrospective chart review for all patients who had undergone LC surgery in a day ward from January 1 to May 5, 2019, including conjunctival congestion or symptoms of conjunctivitis or laboratory microbiology results of ocular secretions to determine the baseline prevalence of conjunctival congestion or conjunctivitis following LC operation.

### Procedural and Environmental Investigations

From May 9 to May 22, 2019, details of the preoperative, intraoperative, and postoperative phases of the care provided to patients undergoing LC were reviewed and the entire procedure was observed. Because patients under general anesthesia were at risk of exposure to keratopathy due to impairment of the protective corneal reflex and ocular muscular flaccidity, these patients' eyes were covered with sterile membranes (polyurethane) without antibacterial ointment at this hospital during surgery. Notably, the size of sterile membranes is too large to match the eyes, so a single sterile membrane was typically cut into several membranes (8 cm in length × 5 cm in width) using non-sterile scissors. We first suspected that those membranes used to cover patients' eyes were contaminated; hence, we randomly collected samples from as many as possible in day surgery and the day ward for incubation, including from the membranes (used to cover our patients' eyes), the non-sterile scissors used for cutting the sterile membrane, laparoscopes (post-sterilization), operating room (OR) air, the anesthesiologists' bare hands, the patients' bare hands, quilt covers, pillowcases, and the cupboards at patients' bedside. Samples were collected as per the standard protocol. The membranes were separately placed into 10 ml of brain heart infusion (BHI) broth. Laparoendoscopic lumens were separately flushed with 10 ml of BHI broth. Samples were collected from non-sterile scissors, anesthesiologists' bare hands, patients' bare hands, quilt covers, pillowcases, and cupboards using moisten sterile cotton swabs, with (0.9% w/v) physiological saline and inoculated on BHI agar plates. OR air samples were collected by sedimentation method, by exposing settle plates with BHI agar left on the air for 30 min (90 mm diameter Petri dishes). All BHI broth and agar plates were incubated for 48 h at 37°C. All colony-forming units (CFUs) were measured and classified using Gram staining and biochemical tests.

### Epidemiologic Studies

**Figure 2:**
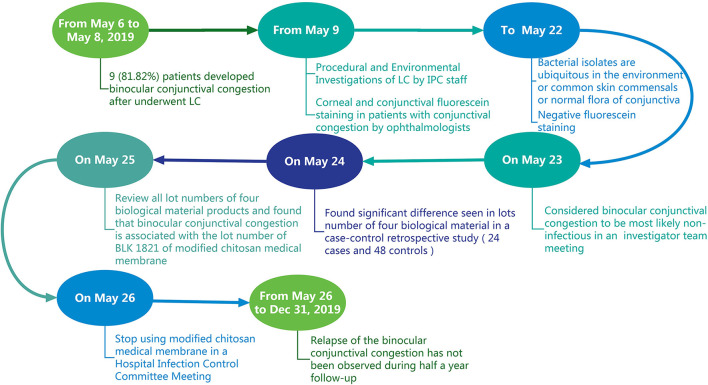
A flow chart of the epidemiology investigation of a cluster of patients who presented with binocular conjunctival congestion after undergoing LC.

A total of 24 patients who underwent LC continued to develop binocular conjunctival congestion until May 23, 2019, so we designed a case-control retrospective study; the 24 patients with binocular conjunctival congestion (cases) after LC was performed from May 6 to May 23, 2019, were matched 1:2 for sex and age (±5 years of age difference) with 48 patients with no binocular conjunctival congestion (controls) who underwent LC from January 1 to May 23, 2019. According to references, data on the following variables were collected: American Society of Anesthesiologists (ASA) score, operators, OR numbers, operative time (from skin incision to skin closure), basic illness (including hypertension, diabetes, and coronary heart disease), blood pressure (on admission), food or drug allergy, intra-abdominal pressure (IAP), and biological material products used and their lot numbers. In addition, we reviewed lot numbers, duration of use, and the number of biological material products used in patients who underwent LC in the day ward. A flow chart of the epidemiology investigation is shown in Figure 2.

### Statistical Analysis

Quantitative data are reported as median (Q25/Q75), statistical analysis was performed using a two-sample *t*-test or Wilcoxon rank-sum test to assess differences in these variables between the two groups, and a *p*-value of <0.05 is considered to show statistical significance.

## Results

### Description of Cases

From January 1 to May 5, 2019, no patients experienced conjunctival congestion or conjunctivitis among 119 consecutive patients who had undergone LC in the day ward. However, the incidence rate of binocular conjunctival congestion after undergoing LC was 80.00% (24/30) from May 6 to May 23, 2019. Case-patients (*n* = 24) had gallstones without suppuration or perforation, and their length of hospital stay did not exceed 24 h in the day ward. All case-patients were aged between 23 and 62 years (median: 47 years) and only presented with binocular conjunctival congestion without secretions, tearing, itching, pain, burning/stinging, photophobia, foreign body sensation, eye discomfort, or decreased vision. Case-patients informed via telephone interviews that binocular conjunctival congestion disappeared naturally within 3–5 days after discharge without the need for antibacterial eye drops; moreover, their surgical incisions were free from signs or symptoms of infection, such as pain or tenderness, localized swelling, redness, or heat. Because there were no conjunctival secretions, there was no chance for culture. Based on negative corneal and conjunctival fluorescein staining results, ophthalmologists concluded that the factor causing binocular conjunctival congestion was most likely non-infectious.

### Procedural and Environmental Investigations

No medical staff had recently suffered from eye diseases, including conjunctival congestion. We collected a total of 218 environmental and bare hand samples to incubate, especially from the remaining membranes after they were cut using non-sterile scissors. After 48 h, no bacteria were isolated from those remaining membranes suspected of being contaminated nor from the laparoscopes. There was a total absence or a low number of bacteria detected in all samples, with the number of colonies ranging from 0 to 2.54 CFU/cm^2^. In addition, all colonies were identified as coagulase-negative staphylococcus (CNS) species, micrococcus species, bacillus species, and diphtheroids, which are ubiquitous in the hospital environment or common skin commensals or normal flora of conjunctiva (Table 1).

**Table 1:** Mean bacterial colony counts and isolated bacteria from 218 samples from day surgery and the day ward.

**Sample**	**No. of samples**	**Mean no. of bacterial colonies**	**Isolated bacteria**
**Day surgery**
Membranes (used to cover patients' eyes)	10	0	None
Laparoendoscopy	10	0	None
Non-sterile scissors	5	1.43 CFU/cm^2^	Bacillus, micrococcus
OR air	73	0.36 CFU^*^	CNS, bacillus, micrococcus
Anesthesiologists' bare hands	10	1.56 CFU/cm^2^	CNS, micrococcus
**Patients in the day ward**
Quilt covers	30	1.14 CFU/cm ^2^	CNS, bacillus, micrococcus
Pillowcases	30	1.78 CFU/cm^2^	CNS, bacillus, micrococcus, diphtheroids
Cupboards	30	2.54 CFU/cm^2^	CNS, bacillus, diphtheroids
Bare hands	20	2.43 CFU/cm^2^	CNS, bacillus

*CFU, colony-forming units; CFU/cm^2^, colony-forming units per square centimeter; CNS, coagulase-negative staphylococcus*.

* *The average number of bacteria per plate was 0.36 CFU. The method of air sampling involved BHI agar plates with a diameter of 9 cm placed in the OR for 30 min to collect airborne bacteria*.

### Case-Control Study

On May 23, 2019, a face-to-face investigator team meeting was held. According to the ophthalmologists' opinion and bacterial isolates from samples, the IPC staff considered that there was no relationship between bacterial infection and binocular conjunctival congestion. Hence, we compared a total of 72 patients who underwent LC from January 1 and May 23, 2019, examining relevant factors between cases and controls (Table 2). There were no significant differences between the two groups in terms of operators, ASA score, OR numbers, operative time, basic illness, blood pressure, food or drug allergy, or IAP. The four biological material products involved in this survey are shown in Table 2; 100% (*n* = 24) of patients in the case group and 97.9% (*n* = 47) of patients in the control group were treated using at least one of the four biological material products. Although there was a significant variation seen among lot numbers of the four biological material products, it was determined that all patients (*n* = 24) with binocular conjunctival congestion after LC were treated with modified chitosan medical membrane (BaiFeiMi, Beijing Bailikang Biochemistries Co., Beijing, China) with a BLK1821 lot number.

**Table 2:** Risk factors in the case and control groups for binocular conjunctival congestion.

**Risk factors**	**Cases (*n* = 24)**	**Controls (*n* = 48)**	***p*-value**
Female sex, *n* (%)	15	30	1.000
Age in years, median (Q25/Q75)	47 (37/53)	46.5 (36/54)	0.830
Operators, *n* (%)			0.842
Operator 1	1	6	
Operator 2	7	13	
Operator 3	1	4	
Operator 4	4	9	
Operator 5	4	7	
Operator 6	7	9	
ASA score, *n* (%)			0.551
1	0	3	
2	21	41	
3	3	4	
Operating room number, *n* (%)			0.061
No. 51	2	6	
No. 52	15	16	
No. 54	7	26	
Operative time (minutes), median (Q25/Q75)	50 (45/78)	60 (46/69)	0.487
**Basic illness**			
Yes	4	9	0.828
**Blood pressure (mmHg), median (Q25/Q75)**			
Diastolic	128 (110/140)	129 (117.75/140)	0.453
Systolic	78 (68/87)	87.5 (73.25/116.5)	0.811
**Food or drug allergy**, ***n*** **(%)**			
Yes	1	4	0.659
IAP (mmHg), *n* (%)^*^			0.221
13	2	1	
14	1	6	
15	16	33	
**Used at least one of four biological material products**, ***n*** **(%)**			
Yes	24 (100.00)	47 (97.92)	1.000
**Used absorbable hemostat**, ***n*** **(%)**			
Yes	13 (54.17)	18 (37.50)	0.178
**Used tissue adhesive produced from enbucrilate**, ***n*** **(%)**			
Yes	13 (54.17)	16 (33.33)	0.089
**Used carboxylated poly (amino acid) polysaccharide medical biological glue**, ***n*** **(%)**			
Yes	5 (20.83)	23 (47.92)	**0.026** ^+^
**Used modified chitosan medical membrane**, ***n*** **(%)**			
Yes	24 (100.00)	39 (81.25)	**0.025** ^+^
**Lot numbers**			
Absorbable hemostat, *n* (%)			**0.000** ^+^
20180801	0	14 (29.17)	
20190101	13 (54.17)	4 (8.33)	
Tissue adhesive produced from enbucrilate, *n* (%)			**0.000** ^+^
217192N1	0	1 (2.08)	
217275N2	0	12 (25.00)	
218083N1	13 (54.17)	3 (6.25)	
Carboxylated poly (amino acid) polysaccharide medical biological glue, *n* (%)			**0.026** ^+^
20180501	0	4 (8.33)	
20190101	0	6 (12.50)	
20190102	4 (16.67)	3 (6.25)	
20190103	1 (4.17)	10 (20.83)	
Modified chitosan medical membrane, *n* (%)			**0.000** ^+^
BLK1818	0	2 (4.17)	
BLK1819	0	13 (27.08)	
BLK1820	0	21 (43.75)	
BLK1821	**24 (100.00)**	3 (6.25)	

* *The electronic medical records system did not record the IAP of 13 patients, resulting in missing data*.

+ *The p-value was considered to show statistical significance (< 0.05)*.

### Intervention Outcome and Follow-Up

**Figure 3:**
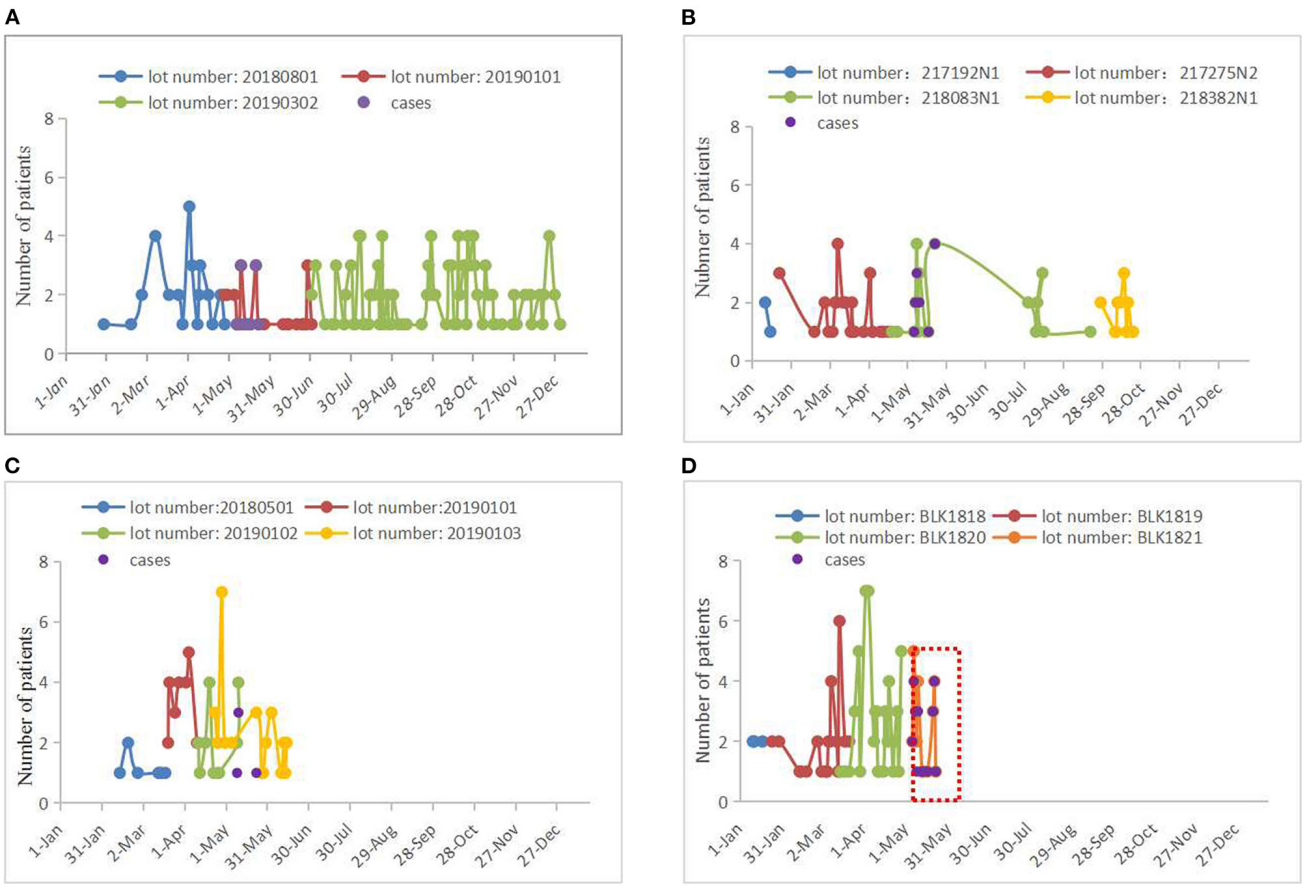
Lot numbers, duration of use, and the number of patients treated with four biological material products in 2019. **(A)** Absorbable hemostat. There were three lot numbers of absorbable hemostat (20180801, 20190101, and 20190302) used by patients who underwent LC in 2019. The case group (*n* = 13 cases) used products with the lot number of 20190101 from May 7 to May 23, 2019, but a total of 32 patients (conjunctival congestion of odds rate: 40.63%) used products from this lot number from April 28 to July 1 (see red line). Last used on December 31, 2019. **(B)** Tissue adhesive produced from enbucrilate. There were four lot numbers of tissue adhesive produced from enbucrilate (217192N1, 217275N2, 218083N1, and 218382N1) used by patients who underwent LC in 2019. The case group (*n* = 13 cases) used products with the lot number of 218083N1 from May 6 to May 22, but a total of 29 patients (conjunctival congestion of odds rate: 44.83%) used products from this lot number from April 19 to September 19, 2019 (see green line). Last used on October 22, 2019. **(C)** Carboxylated poly(amino acid) polysaccharide medical biological glue. There were four lot numbers of carboxylated poly(amino acid) polysaccharide medical biological glue (20180501, 20190101, 20190102, and 20190103) used by patients who underwent LC in 2019. The case group used products with the lot number of 20190102 (*n* = 4 cases) or 20190103 (*n* = 1 case) from May 9 to May 23, 2019, but a total of 18 patients (conjunctival congestion of odds rate: 22.22%) used products with the lot number of 20190102 from April 11 to May 10 (see green line) and a total of 32 patients (conjunctival congestion of odds rate: 3.13%) used products with the lot number of 20190103 from April 23 to June 14 (see yellow line). Last used on June 14, 2019. **(D)** Modified chitosan medical membrane. There were four lot numbers of modified chitosan medical membrane (BLK1818, BLK1819, BLK1820, and BLK1821) used by patients who underwent LC in 2019. The case group (*n* = 24 cases) used products with the lot number of BLK1821 from May 6 to May 23, with a total of 28 patients (conjunctival congestion of odds rate: 85.71%) using products from this lot number from May 6 to May 23. Notably, the period in which all cases appeared overlapped with the period where products with the lot number of BLK1821 were used (see red box). Last used on May 23, 2019.

We first reviewed lot numbers, duration of use, and the number of patients treated with four biological material products during LC in the day ward between January 1, 2019, and May 23, 2019 (Figure 3). We found that only modified chitosan medical membrance with the lot number of BLK1821 was used concurrently with the appearance of cases, from May 6 to May 23, 2019; moreover, 85.7% (24/28) of patients who had used products from the BLK1821 lot were diagnosed with binocular conjunctival congestion after LC (Figure 3D). We therefore surmised that there was a correlation between modified chitosan medical membrance with the lot number of BLK1821 and binocular conjunctival congestion. For this reason, the Hospital Infection Control Committee (HICC) decided to stop the use of the modified chitosan medical membrane product, especially from lot number BLK1821, and established an adverse event reporting system covering conjunctival congestion caused by any biological materials. Relapse of the pseudo-outbreak has not been observed since stopping usage of BLK1821 products for 6 months, and no other procedural changes have been instituted (Figure 3D).

## Discussion

We experienced a cluster of binocular conjunctival congestion after LC from May 6 to May 23, 2019, and conducted an epidemiological investigation to determine the cause of the suspected HAI outbreak, but, after careful analysis, we found that this was not actually an HAI outbreak. Previous studies (11–13) have reported that viruses or bacteria can cause HAI outbreaks, resulting in conjunctivitis and keratoconjunctivitis; however, corneal and conjunctival fluorescein staining results were negative in our case-patients and bacterial isolates from the environment and hands were deemed ubiquitous in the environment or common skin commensals or normal flora of conjunctiva. In addition, case-patients only presented with binocular conjunctival congestion, without other typical signs and symptoms of infection. Hence, we considered that a non-infectious factor most likely had caused the binocular conjunctival congestion in our case-patients. Next, we found that case-patients were likely to have been exposed to biological materials in a case-control retrospective study; notably, all case-patients were exposed to modified chitosan medical membrane with the lot number of BLK1821, and the cluster was ultimately proved to be a pseudo-outbreak caused by this product. Even though binocular conjunctival congestion can resolve naturally without any treatment, its onset may trigger panic among affected patients. Thus, we highlight the urgent need to pay more attention to adverse events of biomaterials in hospitals.

Postoperative adhesion is a common and serious complication in contemporary abdominal surgery, with an estimated incidence of more than 80% (14). Adhesions not only affect the patient's quality of life but also increase the risk of subsequent surgery. To reduce the incidence of postoperative adhesions, barrier-based devices have been investigated widely. In our hospital, surgeons often place a modified chitosan medical membrane in the abdominal cavity before suturing the surgical incision in the abdomen to prevent postoperative adhesion. This product, extracted primarily from shellfish sources, is a highly absorbable biomaterial and can be transformed into colloid in the presence of body fluids, which prevents postoperative adhesion.

Chitosan is the main ingredient of the modified chitosan medical membrane product (BaiFeiMi, Beijing Bailikang Biochemistries Co., Beijing, China), and obtained from the deacetylation of chitin and is a linear cationic polysaccharide that contains copolymers of β-1-4 linked d-glucosamine (GlcN) and N-acetyl-d-glucosamine (GlcNA) units. Various studies have reported that chitosan derivatives possess blood anticoagulation activity as they displayed the capability to delay the clot formation (15–18); other studies (19, 20) have suggested using chitosan as a hemostatic dressing because it contains an abundance of amine groups, which carry a positive charge and attract red blood cells and proteins that carry a negative charge and facilitate coagulation (21). The anticoagulation and coagulation activities of chitosan are linked to its molecular weight (22), concentration (23), and degrees of deacetylation (24); furthermore, the coagulation is proportional to the degree of deacetylation.

Therefore, we speculate that updated lot numbers of the modified chitosan medical membrane product may lead to changes in physicochemical and biochemical properties, thereby causing coagulation disorders, and we also suspect that those products with the BLK1821 lot number may contain an allergen, which leads to an allergic reaction and the release of bioactive mediators, resulting in vascular dilation and increased vascular permeability. Blood vessels of the bulbar conjunctiva are abundant and can be easily detected after coagulation or hyperemia. Hence, binocular conjunctival congestion will likely disappear naturally after the chitosan is metabolized.

Although the presence of a high IAP while undergoing laparoscopic surgery and bacteria may cause conjunctival congestion (25), we predicted that a cluster of binocular conjunctival congestion after LC developed from use of the modified chitosan medical membrane product with the BLK1821 lot number for the following reasons: (1) we recorded negative corneal and conjunctival fluorescein staining results, and bacterial isolates from the environment and hands were ubiquitous in the environment or common skin commensals or normal flora of conjunctiva; (2) there were no significant differences between the cases and controls in terms of IAP and food or drug allergy; (3) the binocular conjunctival congestion disappeared naturally within 3–5 days without any treatment; (4) the lot number of the modified chitosan medical membrane product used to treat patients who underwent LC was switched from BLK1820 to BLK1821 beginning May 6, 2019, and all case-patients were treated with the latter; (5) no relapse of binocular conjunctival congestion in the day ward has been observed since stopping usage of the BLK1821 products.

Currently, although cases of binocular conjunctival congestion caused by chitosan have been reported locally (26–28), no such cases have been reported internationally. Moreover, the National Adverse Drug Reaction Monitoring Center has monitored a suspicious adverse event caused by chitosan in China, and the Food and Drug Administration ordered six manufacturers (including BaiFeiMi, Beijing Bailikang Biochemistries Co., Beijing, China) to suspend the sale of and recall their products and reassessed the impact of chitosan on the blood coagulation system (29).

Although the pseudo-outbreak of binocular conjunctival congestion caused by chitosan derivatives is non-infectious, it still causes panic among patients and concern among medical staff, not to mention that it wastes labor and financial resources. From the twentieth century, chitosan derivatives are widely used in various biomedical applications (30–33). In the last few decades, many researchers have attempted to generate new chitosan derivative-based biomaterials through chemical modification (34); however, few researchers or medical staff pay close attention to adverse events caused by modified chitosan. The IPC is likely to be one of the departments involved in the initial investigation of a suspected HAI outbreak. Timely detection of the cause and termination of clustered events can help to minimize panic among patients and medical staff and save on investigative labor and financial costs.

## Conclusion

In summary, the pseudo-outbreak of binocular conjunctival congestion after LC in our day ward was likely caused by modified chitosan medical membrane products with the lot number of BLK1821. At present, chitosan derivatives are widely used in the field of biomedicine. Owing to the lack of monitoring of adverse reactions of chitosan derivatives in hospitals, it is easy to ignore outbreaks or pseudo-HAI outbreaks caused by them. In health care, chitosan derivatives offer opportunities as well as potential pitfalls. It is essential to pay attention to the monitoring of adverse reactions of chitosan derivatives in hospitals.

## Limitations

Our study had several limitations. First, we did not evaluate the effects of modified chitosan medical membrane products with the lot number of BLK1821 on the activation of clotting factor cascade using activated partial thromboplastin time, prothrombin time, or thrombin time and we did no *t*-test for allergens in these products. We also did not clearly distinguish conjunctival redness from hyperemia. Second, because of limited laboratory testing, we were not able to perform viral testing on patients' samples or the environment. Third, we only focused on patients who underwent LC in the day ward and did not review other patients treated using biomaterials during abdominal and pelvic surgery.

## Data Availability Statement

The original contributions presented in the study are included in the article/supplementary material, further inquiries can be directed to the corresponding authors.

## Ethics Statement

Written informed consent was obtained from the individual(s) for the publication of any potentially identifiable images or data included in this article.

## Author Contributions

SL, QD, and JL: procedural and environmental investigation. XH and CF: statistical analysis. CZ, XM, and YM: data collection for epidemiologic studies. XF and XH data collection for follow-up. SL, CL, and AW: writing-original draft preparation and writing-review and editing. All authors contributed to the article and approved the submitted version.

## Funding

This work was supported by the Key Research and Development Projects of Hunan Province (Nos. 2020SK3027 and 2020SK3028).

## Conflict of Interest

The authors declare that the research was conducted in the absence of any commercial or financial relationships that could be construed as a potential conflict of interest.

## Publisher's Note

All claims expressed in this article are solely those of the authors and do not necessarily represent those of their affiliated organizations, or those of the publisher, the editors and the reviewers. Any product that may be evaluated in this article, or claim that may be made by its manufacturer, is not guaranteed or endorsed by the publisher.
